# Erratum: Impairment of PDGF-induced chemotaxis by extracellular α-synuclein through selective inhibition of Rac1 activation

**DOI:** 10.1038/srep46963

**Published:** 2018-04-06

**Authors:** Taro Okada, Chihoko Hirai, Shaymaa Mohamed Mohamed Badawy, Lifang Zhang, Taketoshi Kajimoto, Shun-ichi Nakamura

Scientific Reports 6: Article number: 37810; 10.1038/srep37810 published online: 11 25 2016; updated: 04 06 2018.

The original version of this Article contained an error in the indexing of the author Shaymaa Mohamed Mohamed Badawy. This error has now been corrected.

